# The combination of SH003 and DTX induces cytotoxic cell infiltration in anti-PD1 resistant lung cancer

**DOI:** 10.1007/s00262-025-04064-6

**Published:** 2025-05-10

**Authors:** Yu-Jeong Choi, Sang-Eun Lee, Daeun Kim, Hae-In Lim, Da Kyung Choi, Bong Kyu Park, Chan-Yong Jeon, Seong-Gyu Ko

**Affiliations:** 1 College of Pharmacy, Natural Products Research Institute, Seoul National University, Seoul, Korea; 2 Department of Science in Korean Medicine, Graduate School, Kyung Hee University, Seoul, Korea; 3 Department of Korean Medicine, Graduate School, Kyung Hee University, Seoul, Republic of Korea; 4 Department of Internal Medicine, College of Korean Medicine, Gachon University, Seongnam, Gyeonggi-do Korea; 5 Department of Preventive Medicine, College of Korean Medicine, Kyung Hee University, Seoul, 02447 Korea

**Keywords:** SH003, Docetaxel, Combination treatment, Anti-PD1 resistance, Lung cancer, EGFR/JAK/STAT3 pathway

## Abstract

The development of therapeutic strategies to overcome resistance to anti-PD1 therapies in lung cancer remains a significant challenge. Based on our recent findings of SH003’s immunomodulatory capabilities, this study investigates the combined effects of SH003 and docetaxel (DTX) as a potential second-line therapy in an anti-PD1-resistant lung cancer model. Our results demonstrate that SH003 and DTX effectively inhibit tumor growth by inducing apoptosis in an anti-PD1-resistant lung cancer LLC1 model, while enhancing the infiltration of cytotoxic CD8^**+**^ T cells and NK cells into the tumor microenvironment (TME), thereby boosting anti-tumor immunity. SH003 also exhibited immunomodulatory effects in an immunosuppressed mouse model, further emphasizing its potential in enhancing immune responses. Notably, the combination treatment significantly inhibits tumor growth by targeting the EGFR/JAK/STAT3 signaling pathway, contributing to the reduction of PD-L1 expression associated with immune evasion. These findings elucidate the dual mechanism of action of the SH003-DTX combination in overcoming resistance through both direct anticancer effects and immune system modulation. Overall, these findings demonstrate that the SH003-DTX combination presents a promising approach for anti-PD1-refractory lung cancer patients, potentially offering new treatment possibilities where current options are limited.

**Supplementary Information:**

The online version contains supplementary material available at 10.1007/s00262-025-04064-6.

## Background

Lung cancer is the second most common cancer, accounting for approximately 20% of all cancer deaths worldwide [1]. The emergence of genetic diagnostics has revolutionized treatment strategies, with targeted therapies serving as first-line approaches for tumors harboring specific mutations in genes such as anaplastic lymphoma kinase (ALK), epidermal growth factor receptor (EGFR), and mesenchymal epithelial transition (MET) [2]. For lung cancers without detectable genetic biomarkers, immunotherapy is recommended based on tumor mutation burden (TMB) or programmed death ligand-1 (PD-L1) expression. However, despite advances in therapies that harness a patient’s immune response, only a small proportion of patients respond well to immunotherapy (~ 20%), and current biomarkers inadequately predict treatment efficacy [3].

Immune checkpoint inhibitors (ICIs) target the protective mechanisms that cancer cells employ to evade immune surveillance. The interaction between programmed death receptor-1 (PD-1) and its ligand PD-L1 inhibits the cytotoxic activity of T cells, enabling unchecked tumor proliferation. While ICIs such as nivolumab, pembrolizumab (anti-PD-1), and atezolizumab (anti-PD-L1) are approved for EGFR wild-type lung cancer treatment, their effectiveness is limited by both intrinsic and acquired resistance. Resistance to immunotherapy emerges through multiple mechanisms, with STAT3 signaling playing a central role. Constitutively activated STAT3 promotes immunosuppression through direct regulation of PD-L1 expression and recruitment of immunosuppressive cells to the tumor microenvironment (TME) [4]. This creates a significant barrier to effective immunotherapy, as STAT3-mediated immunosuppression can persist even when PD-1/PD-L1 interactions are blocked by antibody therapy. Combination regimens incorporating conventional therapies have emerged as promising strategies to overcome these resistance mechanisms [3, 5]. Clinical trials have demonstrated that docetaxel (DTX) chemotherapy, alone or combined with targeted agents, improves overall survival in non-small cell lung cancer (NSCLC) patients previously treated with anti-PD1/L1 therapy, thus representing a viable second-line option [6]. However, optimal combination strategies for ICI-resistant NSCLC remain largely undefined.

SH003, a herbal extract composed of *Astragalus membranaceus* (Am), *Angelica gigas* (Ag), and *Trichosanthes kirilowii Maximowicz* (Tk), has demonstrated remarkable anticancer effects against various malignancies, including breast, prostate, and gastric cancers [7–9]. Recent studies have revealed the synergistic potential of SH003 when combined with DTX, particularly in lung cancer and triple-negative breast cancer [10, 11]. Notably, SH003 not only potentiates the cytotoxic effects of DTX but also mitigates DTX-induced neuropathic pain in mouse models [12]. Additionally, SH003’s immunomodulatory capacity could help reactivate antitumor immune responses [13]. While SH003’s anticancer and immunomodulatory properties have been established, its potential to overcome anti-PD1 resistance through TME modulation remains unexplored. Given the challenges in treating ICI-resistant lung cancer, this study investigates the synergistic effects of SH003 and DTX in anti-PD1-refractory cancer models by simultaneously targeting STAT3-mediated signaling and enhancing immune cell function in the TME.

## Methods

### Syngeneic LLC1 tumor models and drug treatments

**Fig. 1:**
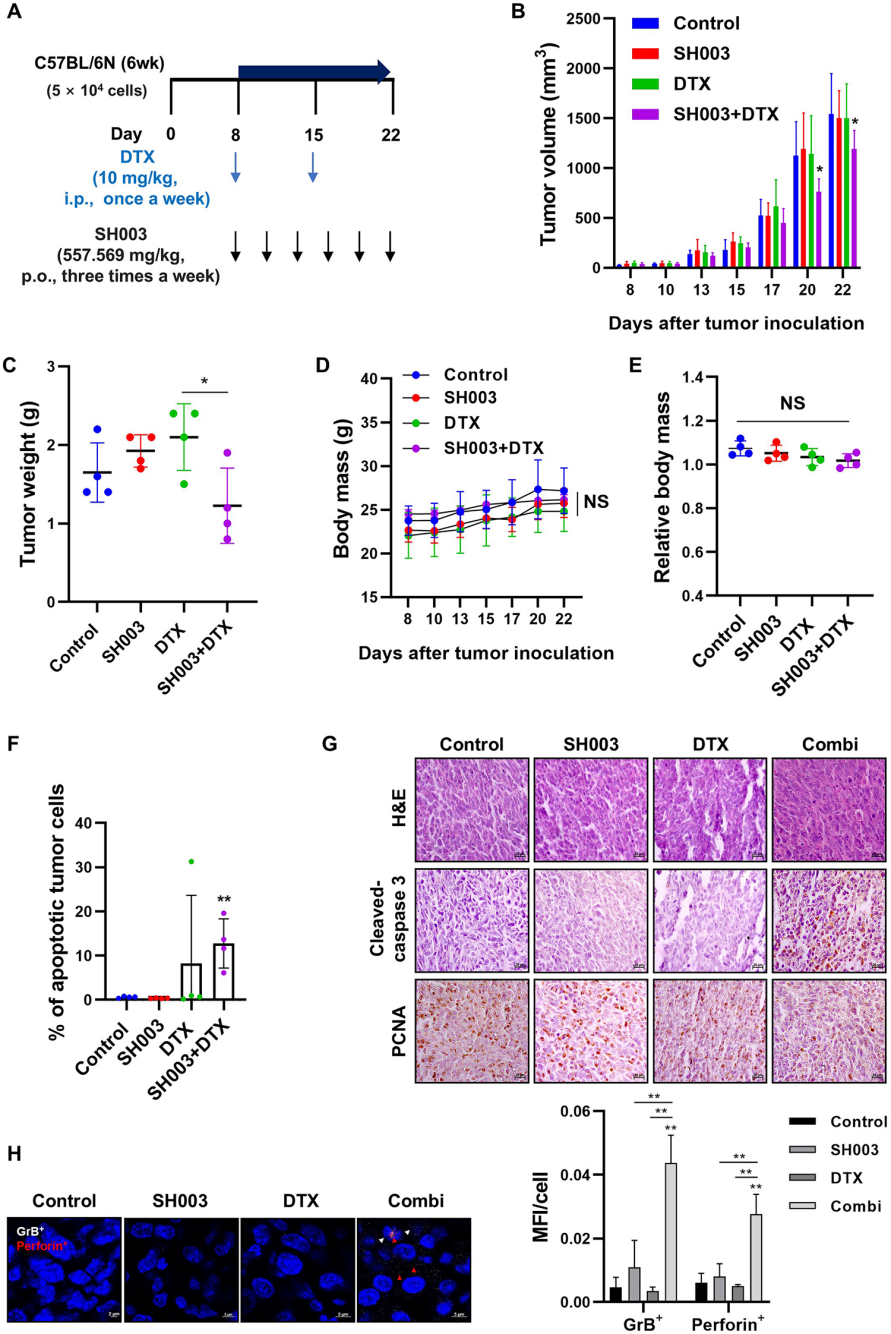
SH003 and DTX combination induces tumor growth suppression via apoptosis in LLC1 syngeneic mouse models **A** Administration schedule of SH003 and DTX. Treatment was initiated when tumors reached approximately 100 mm.^3^ and continued for 2 weeks. **B** and **D** Tumor volume and body mass were measured three times a week. Statistical significance was determined using two-way ANOVA with Bonferroni’s multiple comparisons test. **C** Tumors were harvested post-sacrifice for measurement. Statistical significance was determined using one-way ANOVA with Tukey’s test. **E** Relative body mass was calculated as (Final body mass—Tumor weight)/Initial body mass to account for tumor burden in each treatment group. Data are presented as mean ± SD. **F** Tumors were dissociated into single cells for apoptosis analysis via flow cytometry, stained with Annexin V/7-AAD. ** *p* < 0.01 by Student’s t-test. **G** Tumor samples were fixed in formalin and embedded in paraffin (FFPE) for microscopic analysis (Carl Zeiss, Germany; scale bar: 20 μm). **H** FFPE tumor samples were stained for granzyme B (GrB; red arrows) and perforin (white arrows), with DAPI used as a counterstain. Staining intensity was quantified by mean intensity/DAPI-stained cell count. Statistical significance was determined using two-way ANOVA with Tukey’s test. * *p* < 0.05, ** *p* < 0.01

C57BL/6 N mice [4 weeks old, male] were purchased from the Nara Biotech (Seoul, Korea). The mice were acclimated for two weeks before experiment. LLC1 cells (5 × 10^4^) were implanted subcutaneously in a 1:1 mixture (100 μL) of Matrigel and PBS. When tumors were established after 7 days, the mice were randomly divided into four groups (n = 4) and treated with SH003 (orally) and DTX (intraperitoneally (i.p.)) at indicated doses and times over a period of 2 weeks (Fig. 1A). Tumor volume was measured three times a week using the formula (length x width^2^/2). All animal experiments were approved by the Institutional Animal Care and Use Committee (IACUC) at Kyung Hee University (KHU; KHSASP-23-313) and mice were monitored under specific pathogen-free conditions with a 12/12 h light/dark cycle.

SH003 dosing concentrations were determined by converting the clinical concentrations [10] and prepared by dissolving in 1% ethanol (w/w). DTX was obtained as docetaxel trihydrate (Sigma-Aldrich, MO, USA) and was dissolved in a solution of 10% DMSO, 40% PEG400M, 5% Tween-80, and 45% saline for injection.

### SH003 production and quality control

SH003 extract was manufactured by Hanpoong Pharm and Foods Company (Jeonju, Republic of Korea) under Korea Good Manufacturing Practice (KGMP) standards. The standardized extraction process uses a 30% ethanol solvent to combine *Astragalus membranaceus* (Am), *Angelica gigas* (Ag), and *Trichosanthes Kirilowii Maximowicz* (Tk) roots in a 1:1:1 (w/w) ratio, processed at 100 °C for 3 h. Quality control and standardization are maintained through quantitative HPLC analysis of key bioactive markers: decursin (from Ag) and formononetin (from Am). These compounds serve as primary quality indicators due to their consistent detectability and stability [14]. Each production batch undergoes comprehensive testing according to Korean Pharmacopoeia standards, including heavy metal analysis, microbial limit testing, residual solvent screening, and chromatographic fingerprinting to ensure batch-to-batch consistency. A Certificate of Analysis is generated for each production batch to document compliance with these quality standards.

### Single-cell suspension preparation

Tumors were isolated and dissociated in serum-free DMEM medium containing 1 mg/ml collagenase D (Roche, Basel, Switzerland) and 0.1 mg/mL DNase I (Roche) for 30 min. Tumors and spleens were then filtered through a 70 μm nylon mesh to prepare a single cell suspension and centrifuged at 1500 rpm for 3 min. Cells were treated with RBC lysis buffer to lyse erythrocytes for 3 min, followed by two washes with serum-free medium. Finally, in preparation for flow cytometry, cells were washed with FACS buffer (PBS containing 2% FBS) and filtered through 45 μm strainers.

### Flow cytometry

For apoptosis analysis, cells were stained with Annexin V (BD Biosciences, NJ, USA) and 7-AAD (Sigma-Aldrich, MO, USA) for 15 min at room temperature (RT), respectively. For surface marker staining, cells were incubated with fluorescent-conjugated primary antibodies for 30 min at RT. For intracellular staining of Foxp3, cells were fixed and permeabilized with invitrogen FOXP3/Transcription factor staining set (eBioscience, San Diego, CA) and stained according to the manufacturer’s protocols. All fluorescent staining conditions are summarized in the Table 1. Stained cells were fixed with 2% paraformaldehyde (PFA). For cell surface PD-L1 analysis, cells were treated with SH003 and/or DTX for 24 h. Cells were incubated with the anti-PD-L1 antibody (1:100 dilution in PBS containing 5% BSA) at 37 °C for 30 min. The cells were washed with 1% BSA in PBS and incubated with Alexa Fluor 488-conjugated goat anti-rabbit IgG (Thermo Fisher Scientific, MA, USA) using a 1:250 dilution at 37 °C for 30 min. The cells were then fixed with 0.5% PFA for 10 min. Fluorescence was measured using a CytoFLEX (Beckman Coulter, CA, USA) and analyzed with FlowJo software version 10 (Treestar, OR, USA).

**Table 1:** Fluorescence staining antibody list

Marker	Fluorophore	Catalog number	Dilution	Surface/intracellular
*MHCII*	PE	12-5320-82	1:100	Surface
*CD8*	AF488	53-0081-82	1:100	Surface
*CD25*	PE	12-0251-82	1:100	Surface
CD4	PE-Cy5.5	35-0042-82	1:100	Surface
*Foxp3*	APC	17-5773-82	1:100	Intracellular
PD-1	PE	12-9985-82	1:100	Surface
*Lag-3*	PerCP-EF710	46-2231-82	1:100	Surface
Tim-3	APC	17-5871-82	1:100	Surface
CD3	V450	75-0032-U100	1:100	Surface
CD45R	PE-Cy7	60-0452-U100	1:100	Surface
CD11c	BV650	416-0114-82	1:100	Surface
F4/80	APC-Cy7	25-4801-U100	1:100	Surface
NK1.1	APC	20-5941-U100	1:100	Surface

### Cyclophosphamide (CTX)-induced immunosuppressive mice models

C57BL/6 male mice (7 weeks old) were purchased from Nara biotech (Seoul, Korea) and divided into three groups: control (n = 4; normal), CTX (n = 4; no treatment), and CTX + SH003 (n = 4; SH003 treatment). For immunosuppression, CTX (50 mg/kg) was administrated intraperitoneally on day -4 and 0. On day 0, the CTX + SH003 group mice received SH003 (558 mg/kg) orally once daily for 5 days. During the administration period, body mass was measured and recorded every 2–4 days. At the end of experiment, the spleen was weighed, and the correlation indices were calculated using the following formula:

$$\mathbf{S}\mathbf{p}\mathbf{l}\mathbf{e}\mathbf{e}\mathbf{n}\mathbf{W}\mathbf{e}\mathbf{i}\mathbf{g}\mathbf{h}\mathbf{t}\mathbf{I}\mathbf{n}\mathbf{d}\mathbf{e}\mathbf{x} =\frac{\text{Spleen Weight }(\text{mg})}{\text{Body mass }(\text{g})}$$

### Histology and immunohistochemistry (IHC)

Formaldehyde-fixed and paraffin-embedded (FFPE) tumor tissues were sectioned at 10 μm and stained with hematoxylin and eosin (H&E). For IHC, FFPE tumors were sectioned at 7 μm, deparaffinized, and hydrated. Antigen retrieval was performed with sodium citrate buffer (pH 6.0) for 10 min in a microwave. After cooling, the sections were treated with hydrogen peroxide to inactivate endogenous peroxidase and blocked for 1 h. Primary antibodies were incubated overnight at 4 ℃. The tissues were then incubated with biotinylated secondary antibodies (Vectastain ABC-AP staining kit, Vector Laboratories, CA USA) for 1 h and stained with 3,3′-Diaminobenzidine (DAB). The slides were visualized using a microscope (Carl Zeiss, Germany). The following antibodies were used: Anti-Cleaved-caspase 3, p-STAT3 (Tyr705), and MCL1 (Cell Signaling Technology, MA, USA), CD8, PCNA, and PD-L1 (Abcam, Cambridge, UK), CD206 (Santa Cruz Biotechnology, TX, USA).

### Immunofluorescence (IF)

FFPE tumor tissues were sectioned at 4 μm sections and dried for 45 min at 60 °C. The section slides were deparaffinized, rehydrated, antigen retrieval, and incubated with the fluorescein-conjugated primary antibodies at 37 °C in the dark for 1 h. After incubation, the slides were washed with TBS-T (Tris-buffered saline with 0.1% Tween 20) and mounted for examination. The primary antibodies used were Granzyme B (GrB, PE-Cy5.5), Perforin (APC), and NK1.1 (APC), all purchased from Invitrogen (Thermo Fisher Scientific, MA, USA).

### ELISA

Spleen weight was measured, and the spleens were washed with 1 × PBS to remove excess blood. After two freeze–thaw cycles, the tissues were homogenized in a lysis buffer containing 25 mM Tris–HCl (pH 7.4), 150 mM NaCl, 1 mM EDTA, 1% NP-40, and 5% glycerol, with protease inhibitor (PI) and PMSF, at 4 °C. The homogenates were centrifuged at 16,000 × g for 10 min, and the supernatants were stored at − 80 °C until analysis. Samples were diluted to set the optimum concentration range. Mouse cytokines, including Interleukin 2 (IL-2), Interleukin 6 (IL-6), tumor necrosis factor-alpha (TNF-α), and interferon-gamma (IFN-γ), were detected using DuoSet ELISA Development kits (R&D systems, Seoul, Korea). Cytokine levels were measured at a wavelength of 450 nm using an ELISA reader (Molecular Devices, CA, USA).

### Hematology analysis

In each group, 0.3 mL of blood was collected via cardiac puncture. The blood was placed in evacuated tubes containing K2-EDTA as an anticoagulant (Becton Dickinson, NJ, USA) for hematological analysis. The following hematological parameters were analyzed using a flow cytometry at Biotoxtech Co., Ltd. (Ochang, Korea): White Blood Cell (WBC), Lymphocyte (LYM), and Monocyte (MONO) count.

### Cell culture and cell viability

Lewis lung carcinoma (LLC1) cells, derived from lung tissues of LLC1 tumor-bearing C57BL mice, were purchased from the American Type Culture Collection (ATCC, VA, USA). The cells were maintained in DMEM (WelGENE, Gyeongsan, Korea) containing 10% non-heat-inactivated FBS (JR Scientific, CA, USA) and 1% penicillin/streptomycin solution (WelGENE, Korea).

To measure cell viability, drug-treated cells were incubated for 2 h after the addition of 3-(4,5-Dimethylthiazol-2-yl)-2,5-Diphenyltetrazolium Bromide) (MTT) solution. The absorbance at 570 nm was measured using an ELISA reader (Molecular Devices, CA, USA). The CompuSyn program was used to determine the concentration at which SH003 and DTX exhibit synergistic inhibition.

### Western blotting

Cells were treated with SH003 (100 μg/mL) and DTX (100 nM) for 24 h and lysed with radioimmunoprecipitation assay (RIPA) buffer containing protease and phosphatase inhibitors. Protein concentration was determined using the Bradford assay. Proteins were loaded onto SDS-PAGE gels, transferred to a nitrocellulose membrane, blocked in buffer (1 × PBS with 0.1% Tween-20), and incubated with primary antibodies overnight at 4 °C. After incubation with secondary antibodies for 1 h at room temperature, proteins were detected using an EZ-western detection kit (Dogen-Bio, Seoul, Korea). The primary antibodies used were: PARP, cleaved-caspase 3, EGFR, p-EGFR (Tyr 1068), p-EGFR (Tyr 1173), JAK1, JAK2, p-JAK1 (Tyr1034/1035), p-JAK2 (Tyr1007), STAT3, p-STAT3 (Tyr 705), GAPDH, and MCL1 (Cell Signaling Technology, MA, USA), and PD-L1 (Abcam, Cambridge, UK).

### Transfection

LLC1 cells were seeded at a density of 1 × 10^5^ cells per well. Cells were transiently transfected with pCMV6-AC-GFP control vector (OriGene, PS100010, MD, USA) or STAT3 (OriGene, RC215836, MD, USA) using Lipofectamine 3000 reagent (Invitrogen, CA, USA) for 24 h. Following 24 h of SH003 and/or DTX treatment, the cells were harvested for further analysis.

### ***Correlation between STAT3 expression and Activated CD8***^+^***T Cells using TISIDB database***

To investigate the correlation between STAT3 expression and the abundance of activated CD8^**+**^ T cells in lung adenocarcinoma (LUAD), we utilized the TISIDB database (http://cis.hku.hk/TISIDB/). A scatter plot was generated to visualize the relationship between STAT3 expression levels and the abundance of activated CD8^**+**^ T cells. Spearman’s correlation coefficient (r) was calculated, and the corresponding p-value was included to assess the statistical significance of the observed correlation.

### Statistical analysis

Data are presented as mean ± standard deviation (SD). Statistical analysis was performed using GraphPad Prism software 8.0.2 (GraphPad Software, CA, USA). *p-*values were determined using one-way ANOVA with multiple comparison test. Pearson’s correlation coefficient (r) was also analyzed using GraphPad Prism software 8.0.2.

## Results

### SH003 and DTX inhibit an anti-PD1-resistant lung cancer tumor model by inducing apoptosis

To explore the potential efficacy of SH003 and docetaxel (DTX) in overcoming resistance to anti-PD1 therapy, we employed the LLC1 lung cancer syngeneic mouse model, which is known to be refractory to anti-PD1 treatment. The combination of SH003 and DTX was administered for 2 weeks, following the regimen outlined in Fig. 1A. This combination significantly inhibited tumor growth compared to both control and monotherapy groups (Fig. 1B) and resulted in a reduction in tumor weight relative to the DTX-only group (Fig. 1C). Importantly, no significant toxicity was observed in the mice during treatment (Fig. 1D). Further analysis of relative body mass showed stable values across all groups (Control: 1.07, SH003: 1.05, DTX: 1.03, Combination: 1.02), confirming that the treatments were well-tolerated even when accounting for tumor burden (Fig. 1E). Apoptosis has been identified as a critical mechanism underlying the effects of SH003 and DTX in previous studies [10, 11]. Consistent with these findings, we observed a significant increase in apoptotic tumor cells in the combination treatment group (Fig. 1F). Immunohistochemical analysis also showed increased expression of the apoptosis marker cleaved-caspase 3 and decreased expression of the proliferation marker PCNA in tumor tissues following combination treatment (Fig. 1G). These data suggest that apoptosis plays a crucial role in the antitumor effects of the combination treatment. Given the role of immune evasion in resistance to immunotherapy [15], we next sought to determine whether the observed induction of apoptosis was linked to immune cell activity in the TME. Cytotoxic lymphocytes, including T cells and natural killer (NK) cells, are key mediators of tumor suppression, acting through the release of signaling molecules such as perforin and granzyme B (GrB). Perforin disrupts the cell membrane of target cells, allowing GrB to enter and trigger the caspase-mediated cell death cascade [16, 17]. Immunofluorescence staining revealed the translocation of both GrB and perforin into tumor cells following combination treatment (Fig. 1H), indicating the activation of a perforin/GrB-dependent apoptotic pathway driven by immune cells. Overall, SH003 and DTX exhibit enhanced antitumor activity, primarily by inducing apoptosis through both direct and immune-mediated mechanisms. The observed modulation of the perforin/GrB pathway suggests a potential for this combination treatment to enhance immune responses in anti-PD1-refractory lung cancer.

### SH003 and DTX combination enhances cytotoxic immune cell infiltration in LLC1 tumors

 Given the observed association between combination treatment and immune cell-mediated tumor suppression, we further investigated whether this effect was due to the induction of immune activation. Specifically, we examined the impact on T-cell exhaustion and immunosuppressive regulatory T cells (Tregs), both of which are known to promote resistance to immune checkpoint inhibitors (ICIs). We evaluated the expression levels of inhibitory checkpoints LAG-3, TIM-3, and PD-1, which are typically upregulated during T-cell depletion [18], as well as the intratumoral CD4^**+**^CD25highFoxp3^**+**^ Treg population [19]. Notably, the combination treatment did not restore exhausted T-cell function and had no effect on Treg cell suppression (Supplementary Fig. 1).

**Fig. 2:**
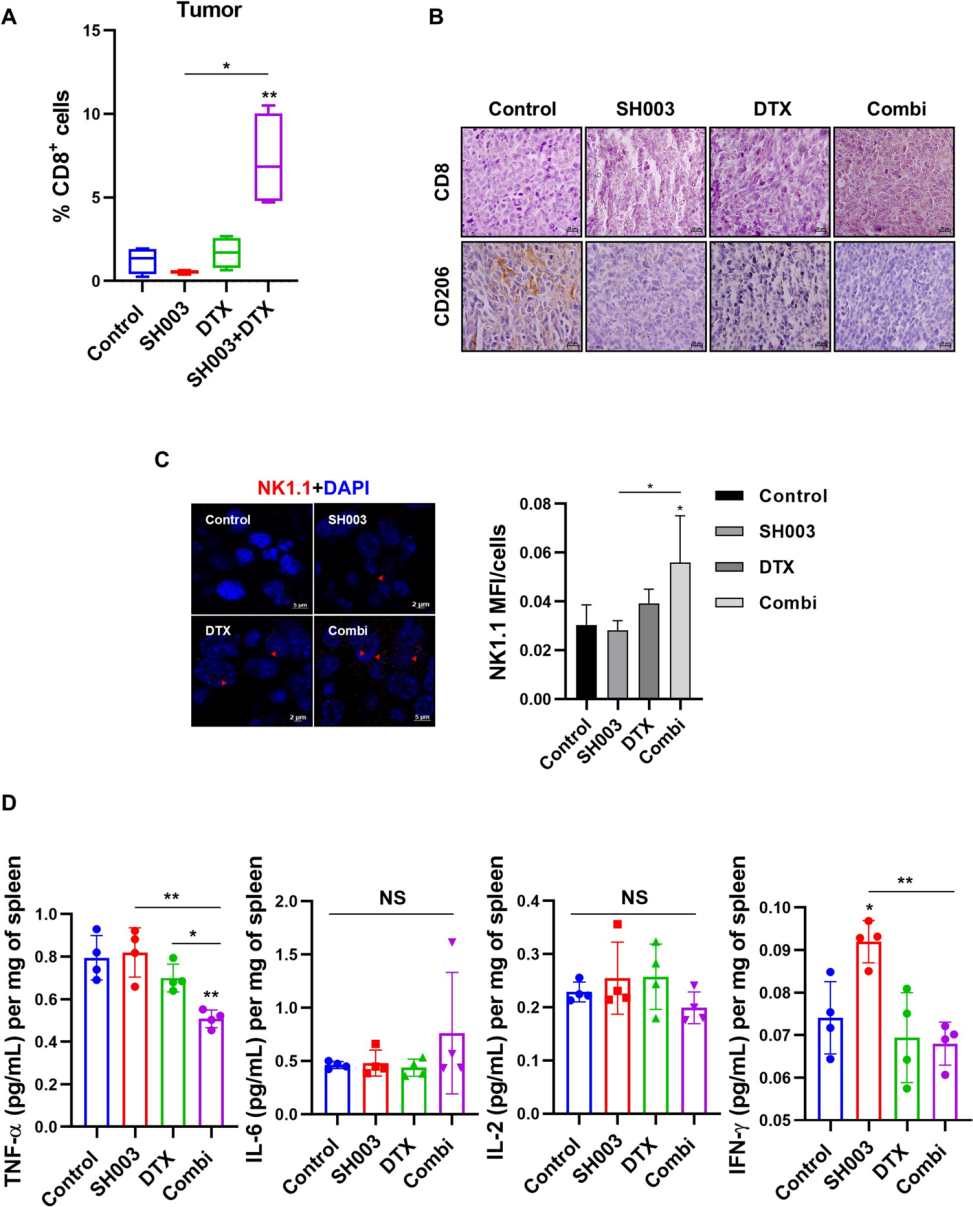
SH003 and DTX treatment enhances immune response by increasing tumor-infiltrating immune cells. **A** Flow cytometry analysis of CD8^**+**^ cells within the tumor. Statistical significance was determined using one-way ANOVA with Tukey’s test and Student’s t-test. **B** IHC analysis of tumor tissue. Scale bar: 20 μm. **C** FFPE tumor samples were stained for NK1.1 (NK cell marker; red arrows) and counterstained with DAPI. Staining intensity was quantified by the mean intensity per cell count. Statistical significance was determined using one-way ANOVA with Bonferroni’s multiple comparisons test. **D** Spleen tissues were homogenized, and cytokine levels were measured in the supernatants at 450 nm absorbance. * *p* < 0.05, ** *p* < 0.01 by one-way ANOVA with Tukey’s test

In addition to immune cell function, enhancing the infiltration of immune cells into tumors can result in a robust antitumor immune response, often referred to as a “hot tumor” phenotype [20, 21]. Using the antibody panel detailed in Table 1, we assessed the effect of combination treatment on immune cell infiltration into tumor tissue. Our analysis revealed a significant increase in intratumoral CD8^**+**^ cells following combination treatment (Fig. 2A and B), indicating enhanced cytotoxic T-cell infiltration into the TME. We also investigated other immune cell populations within the TME. The levels of CD206, a marker of immunosuppressive M2-polarized macrophages, and CD11b^**+**^ myeloid-derived suppressor cells (MDSCs), which contribute to tumor progression, were significantly reduced in the combination treatment group (Fig. 2B, bottom row). Conversely, expression of NK1.1, a marker for natural killer (NK) cells, was elevated, suggesting enhanced NK cell-mediated antitumor activity (Fig. 2C). These results demonstrated that combination treatment modulates specific immune components within the TME, showing selective enhancement of cytotoxic immune cell infiltration and reduction of immunosuppressive M2 macrophages and MDSCs. To further evaluate antitumor immune activity, we analyzed cytokine production in the spleen. Cytokines such as IL-6, TNF-α, IL-2, and IFN-γ are typically secreted by activated T cells and serve as markers of the antitumor immune response [22]. Interestingly, the combination treatment did not alter IL-6 or IL-2 levels but resulted in decreased secretion of IFN-γ and TNF-α compared to either DTX or SH003 alone (Fig. 2D). While these cytokines increase during the early stages of the T-cell-mediated immune response, their levels can decrease during sustained tumor suppression. Prolonged overexpression of proinflammatory cytokines may convert them into tumor-promoting factors [23, 24]. Thus, our data suggest that the combination treatment may prevent excessive inflammation while maintaining antitumor immunity, rather than initiating an acute inflammatory response. Collectively, the combination treatment demonstrated selective enhancement of cytotoxic immune cell populations, particularly CD8^**+**^ T cells, while maintaining their functional capacity as shown by perforin/granzyme B activity. These findings indicate that the combination treatment promotes selective immune cell recruitment and activation rather than broad immune system stimulation.

### SH003 exhibits immunomodulatory effects in an immunosuppressed mouse model

**Fig. 3:**
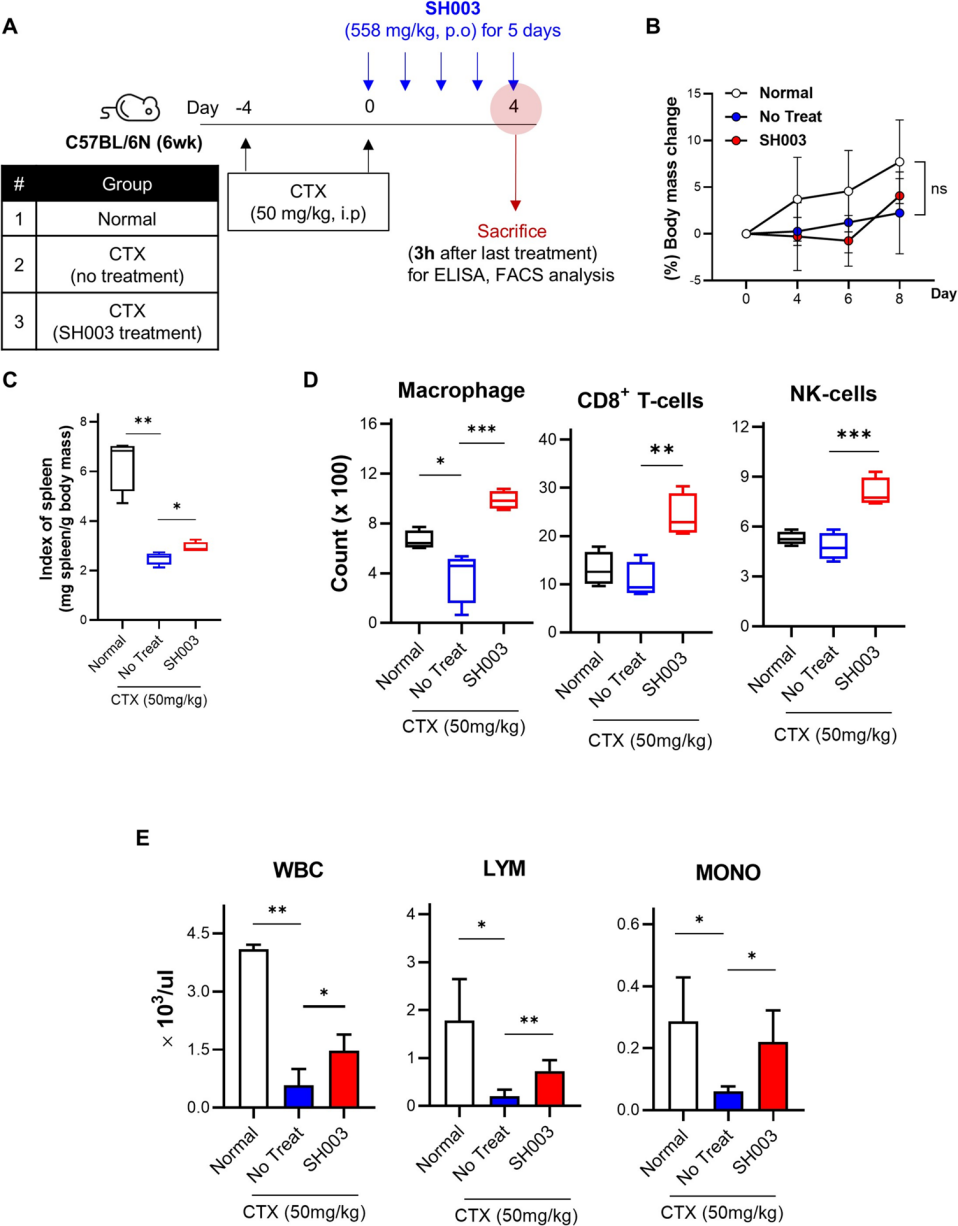
SH003 restores immune functions in CTX-induced immunosuppressive mice. **A** Diagram showing the induction of the immunosuppressive mouse model and the administration schedule of SH003. CTX was used to induce immunosuppression, followed by SH003 treatment. **B** body mass of mice was measured and recorded every 2–4 days. ns: not significant; No treat: no treatment **C** Spleen index was calculated to assess spleen size relative to body mass. **D** Flow cytometry analysis of absolute cell numbers in the spleen. * *p* < 0.05, ** *p* < 0.01, *** *p* < 0.001 by one-way ANOVA with Tukey’s test. **E** Blood samples were collected via cardiac puncture, and hematological analysis was performed using flow cytometry (Biotoxtech Co., Ltd., Ochang, Korea). * *p* < 0.05, ** *p* < 0.01 by one-way ANOVA with Tukey’s test

Previous studies have shown that DTX enhances tumor-infiltrating lymphocytes (TILs) in lung cancer, increasing T cell and NK cell populations [25]. DTX combined with immunotherapy increases antitumor efficacy through immune cell infiltration [26]. However, DTX alone often shows limited efficacy in resistant patients due to insufficient T-cell infiltration [27]. To investigate SH003’s potential as a DTX adjuvant under immunosuppressive conditions, we employed a cyclophosphamide (CTX)-induced immunosuppression model (Fig. 3A). CTX specifically depletes lymphocyte populations, creating an immunosuppressed state that shares key characteristics with anti-PD1-resistant conditions, particularly the reduced lymphocyte populations that limit treatment efficacy [28]. SH003-treated mice showed a significant increase in spleen index compared to the CTX-only group, indicating immune system expansion without affecting body mass (Fig. 3B and C). Flow cytometry analysis using fluorescence-conjugated antibodies (Table 1) showed that SH003 treatment significantly increased macrophage, CD8^**+**^ T cell, and NK cell populations in the spleen (Fig. 3D). Furthermore, SH003 elevated white blood cell (WBC), lymphocyte, and monocyte levels in the blood (Fig. 3E). These findings suggest SH003’s potential to enhance cancer therapy efficacy by modulating immune cell populations under immunosuppressive conditions.

### Combination treatment of SH003 and DTX induces apoptosis in LLC1 cells

**Fig. 4:**
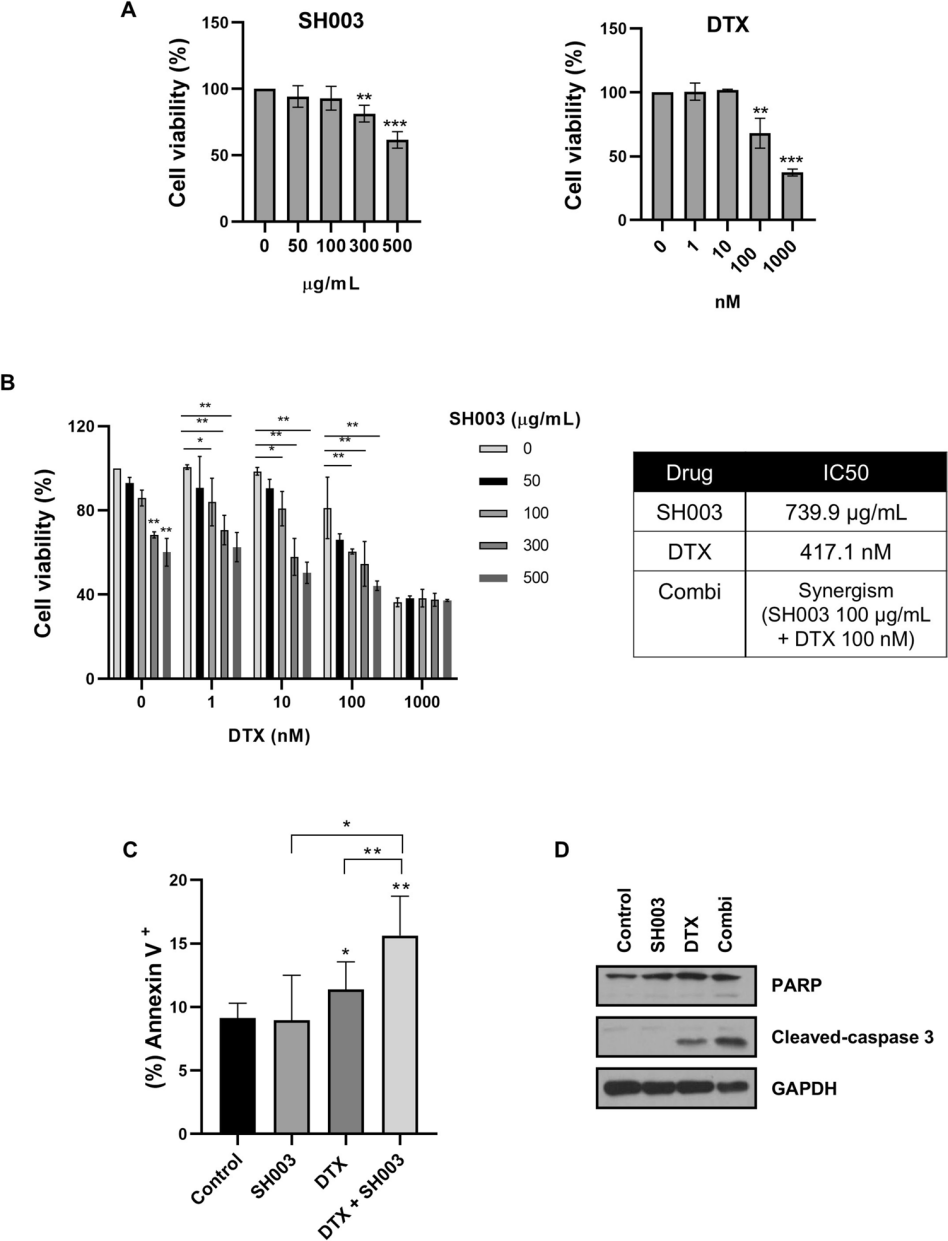
SH003 and DTX co-treatment synergistically induces apoptosis in LLC1 cells. **A** LLC1 cells were treated with SH003 or DTX for 48 h. Cell viability was measured using MTT assay and data were normalized to control cells. Statistical significance was determined using Student’s t-test. **B** Following 48 h of combination treatment, the IC50 values for each drug were calculated using the Prism software, and the combination doses with synergistic effects were determined using the CompuSyn software. **C** LLC1 cells were treated with the combination for 24 h, and apoptosis was analyzed by flow cytometry. Statistical significance: * *p* < 0.05, ** *p* < 0.01 by one-way ANOVA with Tukey’s test. **D** Western blot analysis of cells exposed to the combination treatment for 24 h to detect protein expression levels associated with apoptosis

To further elucidate the molecular mechanisms underlying the apoptotic effects observed in vivo, we examined the direct impact of the combination treatment on LLC1 cells in vitro. Treatment with SH003 and DTX reduced cell viability in a dose-dependent manner, with the combination demonstrating synergistic effects (CI = 0.45) (Fig. 4A and B). At synergistic concentrations (SH003: 100 μg/mL, DTX: 100 nM), the combination treatment induced a marked increase in apoptotic cell populations compared to SH003 or DTX alone (Fig. 4C). Mechanistically, we observed enhanced cleavage of pro-apoptotic proteins PARP and caspase-3 following combination treatment (Fig. 4D), indicating that the combination treatment effectively induced apoptosis in LLC1 cells. These findings support our in vivo observations that apoptosis is a key mechanism underlying the enhanced antitumor effects of the combination treatment.

### SH003 and DTX inhibit EGFR/STAT3/PD-L1 signaling to promote apoptosis in anti-PD1-resistant LLC1 cells

**Fig. 5:**
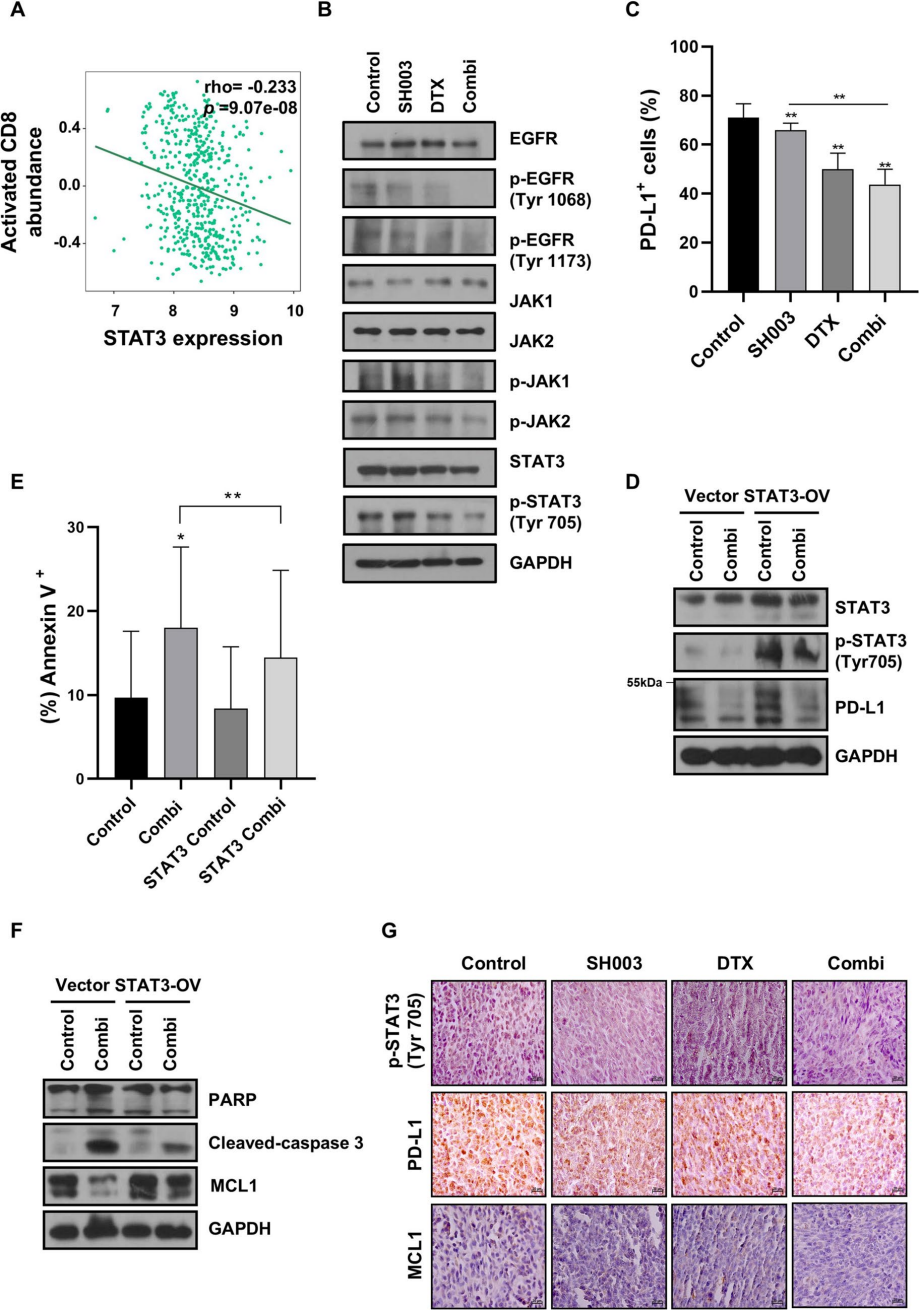
SH003 and DTX induces apoptosis in LLC1 cells by inactivating the STAT3/PD-L1 signaling pathway. **A** Analysis of the interaction between STAT3 expression levels and the abundance of activated CD8^**+**^ T cells in human lung cancer using the TISIDB database. **B** Western blot analysis of cells exposed to the combination treatment for 1 h. **C** Flow cytometry measurement of PD-L1-positive LLC1 cells treated with the combination of SH003 and DTX for 24 h. Statistical significance was determined using one-way ANOVA with Bonferroni’s test. **D**–**F** LLC1 cells were transfected with the p-CMV6-STTA3 vector and treated with the combination for 24 h. Apoptosis induction was analyzed by flow cytometry. Statistical significance was determined using one-way ANOVA with Bonferroni’s test. Western blot analysis was performed to examine the STAT3/PD-L1 and apoptosis pathways. **G** Tumor IHC analysis of the STAT3/PD-L1 pathway and the apoptotic marker MCL1. Scale bar: 20 μm

EGFR, frequently mutated in lung cancers, serves as a pivotal driver of cancer growth and contributes to resistance against targeted anticancer drugs. The JAK/STAT3 signaling pathway, activated by EGFR, promotes immunosuppression and resistance to anti-PD1 therapies. Analysis of the TISIDB database revealed a negative correlation between STAT3 expression and activated CD8^**+**^ T cell infiltration in lung adenocarcinoma (LUAD) (Fig. 5A), suggesting STAT3’s role in immune evasion. Given STAT3’s role in anti-PD1 resistance, we investigated whether the combination treatment targets this pathway in anti-PD1 refractory LLC1 cells. The combination treatment significantly inhibited phosphorylation of EGFR/JAK/STAT3 (Fig. 5B). Additionally, expression of PD-L1, a STAT3-regulated protein, decreased significantly following combination treatment (Fig. 5C), indicating suppression of immune evasion mechanisms. To confirm STAT3’s role in treatment-induced cell death, we evaluated the apoptotic response in STAT3-overexpressing cells. STAT3 overexpression prevented combination treatment-induced apoptosis, as evidenced by reduced cleavage of PARP and caspase-3 (Fig. 5E and F). The combination treatment reduced expression of PD-L1, a STAT3-regulated protein involved in immune evasion, and MCL1, a STAT3-regulated anti-apoptotic protein (Fig. 5D and 5F). Importantly, these effects were recapitulated in tumor tissues, where treatment reduced both STAT3 activation and expression of downstream signaling proteins (Fig. 5G). Collectively, our results reveal that the SH003 and DTX combination effectively targets both tumor-intrinsic signaling pathways and immune-mediated mechanisms, providing a dual approach to overcoming anti-PD1 resistance through simultaneous modulation of the EGFR/STAT3/PD-L1 axis and enhancement of immune-mediated cytotoxicity in the TME.

## Discussion

**Fig. 6:**
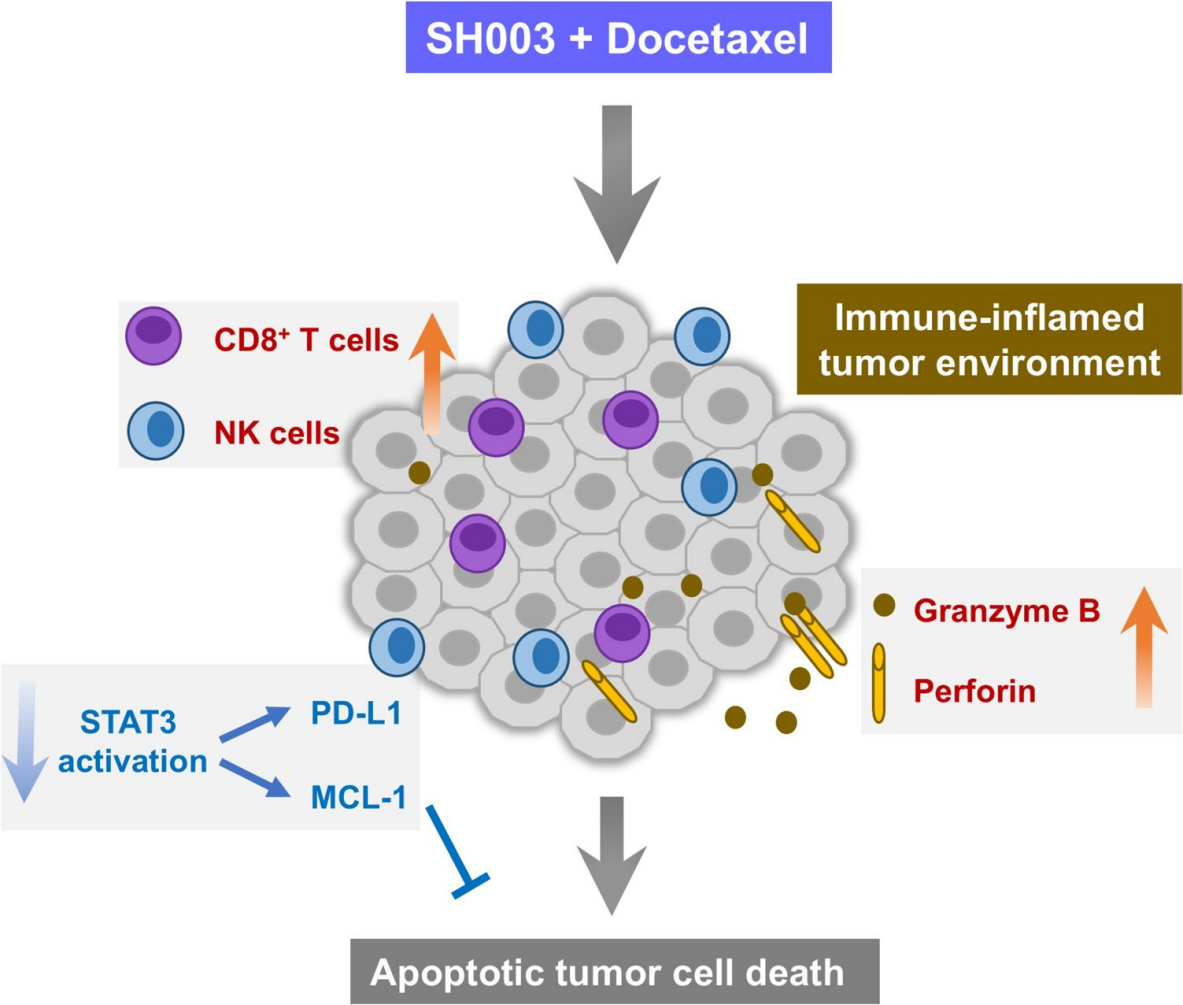
Mechanism by which SH003 and DTX suppress anti-PD1-refractory lung cancer. SH003/DTX combination enhances anti-tumor immunity through cytotoxic immune cell infiltration and promotes apoptosis via granzyme B/perforin secretion and STAT3 inhibition

We investigated the synergistic effects of SH003 and DTX in overcoming resistance mechanisms in an anti-PD1-resistant lung cancer model. The combination enhanced intratumoral antitumor immune responses by increasing cytotoxic immune cell infiltration, particularly CD8^**+**^ T cells and NK cells, while reducing immunosuppressive M2 macrophages in the TME. Importantly, the combination inhibited tumor growth and reduced PD-L1 expression by targeting the EGFR/JAK/STAT3 signaling pathway, demonstrating potential to overcome anti-PD1 therapy resistance (Fig. 6).

For patients with anti-PD1-refractory lung cancer, standard treatment often combines immune checkpoint inhibitors (ICIs) with conventional chemotherapy, such as DTX. While DTX enhances antitumor immune responses through increased tumor antigen presentation and reduced immunosuppressive cell populations, most patients still fail to overcome resistance due to insufficient T-cell infiltration and a highly immunosuppressive TME [26, 27, 29]. Our findings demonstrate that SH003 complements DTX by inducing immune cell-mediated apoptosis through the perforin/granzyme B pathway and reconstituting the TME to enhance antitumor immunity. Notably, in immunosuppressive models where immune attacks typically fail, SH003 increased immune cell levels in the spleen and blood, effectively converting an immunosuppressive environment to an immune-responsive one. This comprehensive immune activation by SH003 supports its potential as an effective DTX adjuvant, potentially improving ICI-based therapy outcomes in resistant lung cancers.

Our findings demonstrated enhanced cytotoxic cell infiltration and reduced immunosuppressive myeloid populations following combination treatment, but we observed no significant changes in Treg populations and T cell exhaustion status. This result indicated that the combination treatment appears to primarily recruit and enhance functional CD8^**+**^ T cells, as evidenced by increased perforin/granzyme B activity, demonstrating that infiltrating CD8^**+**^ T cells retain their cytotoxic functionality despite the presence of Tregs. Recent studies support this selective immune enhancement, where successful immunotherapy can be achieved through targeted enhancement of specific immune populations without broadly altering the entire immune landscape [30–32]. Also, this apparent paradox may actually reflect distinct temporal and spatial dynamics of immune response modulation. We evaluated all immunological state in sustained antitumor response. Our cytokine analysis revealed decreased levels of IFN-γ and TNF-α in the combination treatment group at this tumor suppression time point, suggesting the establishment of a sustainable antitumor immune environment rather than an acute, potentially harmful inflammatory state. Collectively, our data highlights that combination’ tumor suppression response induce selective immune cell recruitment and balanced immune response. Such balance is increasingly recognized as crucial for durable therapeutic responses in cancer immunotherapy, particularly in the context of combination treatments targeting immune checkpoint resistance.

Tumor-infiltrating lymphocytes (TILs), particularly cytotoxic T cells, serve as critical indicators of therapeutic response, with higher TIL levels correlating with improved clinical outcomes [33]. Gene and immune system interaction analysis revealed that higher STAT3 expression correlated with a decrease in activated CD8^**+**^ T cells in lung cancer, suggesting STAT3’s role in immune suppression. This aligns with recent findings demonstrating that STAT3 activation contributes to ICI resistance by promoting immune dysfunction, and that STAT3 inhibition combined with immunotherapy effectively overcomes treatment resistance [34–36]. Notably, SH003 has previously demonstrated STAT3 inhibitory effects in non-small cell lung cancer (NSCLC) while synergistically enhancing DTX efficacy (11, 37). In our study, the combination treatment effectively regulated multiple nodes in the STAT3 signaling network, as evidenced by inhibition of both upstream regulators (p-EGFR) and downstream effectors (MCL1, PD-L1). This comprehensive modulation of the STAT3 pathway occurred concurrently with enhanced intratumoral CD8^**+**^ T cell populations and reduced PD-L1 levels. Although the direct causal relationship between STAT3 inhibition and enhanced immune cell infiltration requires further investigation, our findings suggest that targeting the EGFR/STAT3/PD-L1 axis could be a pivotal strategy for overcoming resistance to anti-PD1 therapy. Moreover, the modulation of TIL levels by the SH003 and DTX combination treatment may serve as a potential biomarker to assess treatment effectiveness and improve drug response in ICI treatment strategies for anti-PD1 refractory lung cancer patients.

While our study demonstrates novel tumor suppressive mechanisms of the SH003/DTX combination in overcoming anti-PD1 resistance, several limitations warrant consideration. Although informative, our anti-PD1-refractory mouse tumor model cannot fully recapitulate the complexity of human tumors, necessitating clinical validation of the combination’s efficacy and safety. Additional investigation is needed to elucidate the precise molecular mechanisms underlying enhanced immune cell infiltration and TME regulation. Future studies should evaluate this combination treatment across different ICI resistance models to determine whether its effects are specific to anti-PD1 resistance or applicable to other forms of immunotherapy resistance. Also, our single-timepoint analysis at tumor suppression represents a limitation that may have missed earlier dynamic changes in immune cell populations. Future studies incorporating temporal analysis of immune cell dynamics could provide valuable insights into the relationship between effector and regulatory immune populations, potentially supporting a more comprehensive understanding of the combination treatment’s immunomodulatory mechanisms.

## Conclusion

This study demonstrates that the combination of SH003 and DTX effectively suppresses anti-PD1 refractory lung cancer by modulating the immune environment and enhancing the infiltration of cytotoxic immune cells. The combination treatment inhibited the EGFR/JAK/STAT3 signaling pathway and reduced PD-L1 expression, concurrent with increased immune-mediated tumor cell death through the perforin/granzyme B pathway. These findings reveal potential therapeutic mechanisms involving both immune modulation and signaling pathway regulation, highlighting SH003 as a valuable adjunct to DTX therapy. This combination strategy offers new therapeutic possibilities for patients with anti-PD1 resistance, potentially expanding treatment options in refractory lung cancer.

## Supplementary Information

Below is the link to the electronic supplementary material.

Supplementary file1 (DOCX 169 kb)

## Data Availability

No datasets were generated or analysed during the current study.
