# Supplementary material for: The combination of SH003 and DTX induces cytotoxic cell infiltration in anti-PD1 resistant lung cancer
Source: Cancer Immunol Immunother. 2025 May 10;74(7):198. doi: 10.1007/s00262-025-04064-6 (PMC12065697; doi:10.1007/s00262-025-04064-6)
Supplement: Supplementary file 1 — Supplementary file1 (DOCX 169 kb) [file 262_2025_4064_MOESM1_ESM.docx]

**The combination of SH003 and DTX Induces Cytotoxic Cell Infiltration in Anti-PD1 Resistant Lung Cancer**

**Yu-Jeong Choi ^1^, Sang-Eun Lee ^2^, Daeun Kim ^2^, Hae-In Lim ^2^, Da Kyung Choi ^3^, Bong Kyu Park ^3^, Chan-Yong Jeon ^4^, Seong-Gyu Ko ^5, *^**

^1^ Natural Products Research Institute, College of Pharmacy, Seoul National University, Seoul, Korea

^2^ Department of Science in Korean Medicine, Graduate School, Kyung Hee University, Seoul, Korea

^3^ Department of Korean Medicine, Graduate School, Kyung Hee University, Seoul, Republic of Korea;

^4^ Department of Internal Medicine, College of Korean Medicine, Gachon University, Gyeonggi-do, Korea

^5^ Department of Preventive Medicine, College of Korean Medicine, Kyung Hee University, Seoul, Korea

* Correspondence should be addressed to Seong-Gyu Ko; [epiko@khu.ac.kr](mailto:epiko@khu.ac.kr)

Department of Preventive Medicine, College of Korean Medicine, Kyung Hee University, Seoul 02447, Korea


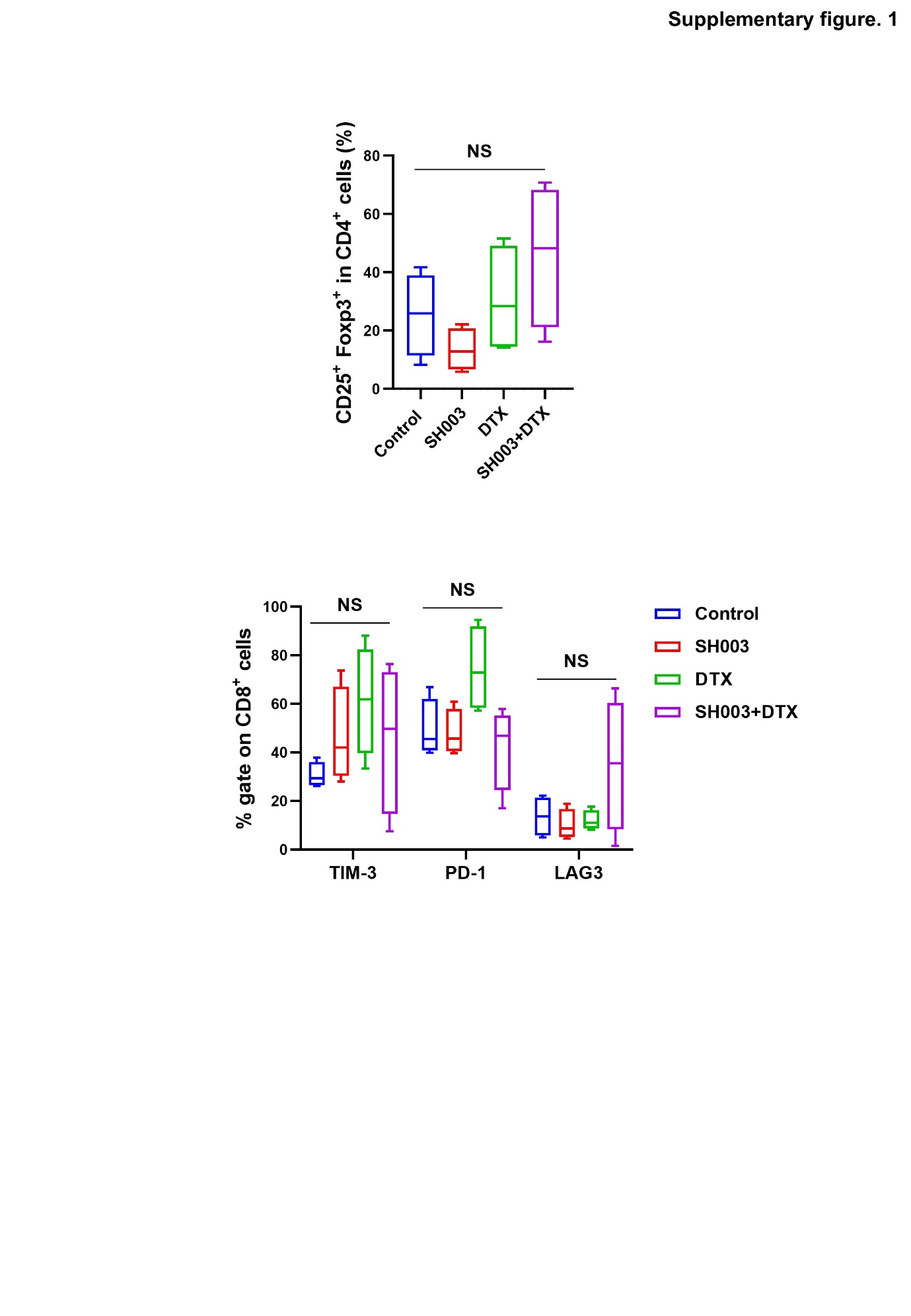


**Fig. S1. Analysis of T cell exhaustion and regulatory T cell populations across treatment groups.** Expression levels of regulatory T cell (CD25^+^/Foxp3^+^/CD4^+^) and exhaustion markers (TIM-3, PD-1, and LAG3) on CD8^+^ T cells shown as percentage of cells expressing each marker. No significant (NS) differences were observed between groups.
